# Controllably releasing long-lived quantum memory for photonic polarization qubit into multiple spatially-separate photonic channels

**DOI:** 10.1038/srep33959

**Published:** 2016-09-26

**Authors:** Lirong Chen, Zhongxiao Xu, Weiqing Zeng, Yafei Wen, Shujing Li, Hai Wang

**Affiliations:** 1 The State Key Laboratory of Quantum Optics and Quantum Optics Devices, Collaborative Innovation Center of Extreme Optics, Institute of Opto-Electronics, Shanxi University, Taiyuan, 030006, People’s Republic of China

## Abstract

We report an experiment in which long-lived quantum memories for photonic polarization qubits (PPQs) are controllably released into any one of multiple spatially-separate channels. The PPQs are implemented with an arbitrarily-polarized coherent signal light pulses at the single-photon level and are stored in cold atoms by means of electromagnetic-induced-transparency scheme. Reading laser pulses propagating along the direction at a small angle relative to quantum axis are applied to release the stored PPQs into an output channel. By changing the propagating directions of the read laser beam, we controllably release the retrieved PPQs into 7 different photonic output channels, respectively. At a storage time of *δt* = 5 *μs*, the least quantum-process fidelity in 7 different output channels is ~89%. At one of the output channels, the measured maximum quantum-process fidelity for the PPQs is 94.2% at storage time of *δt* = 0.85 *ms*. At storage time of 6 ms, the quantum-process fidelity is still beyond the bound of 78% to violate the Bell’s inequality. The demonstrated controllable release of the stored PPQs may extend the capabilities of the quantum information storage technique.

Quantum networks (QNs), comprising of many quantum nodes and quantum channels, provide a crucial platform to perform scalable quantum information processing^1^,^2^. Quantum nodes are used for processing and storing quantum information (qubits). Quantum memory is an essential ingredient for long distance quantum key distribution^3^ and quantum secure direct communication^4^,^5^,^6^,^7^,^8^, entanglement distribution and teleportation^9^,^10^,^11^,^12^. Quantum channels are used for transporting quantum information between different nodes^1^. Flying photon qubits are good carriers of quantum information since they travel fast and weakly interact with environment^1^,^2^. Cold atomic ensembles are promising matter nodes^1^,^2^ since long-lived and/or efficient quantum memories for single photon or photon qubits can be achieved via spontaneous Raman scattering^13^,^14^,^15^,^16^, dynamic electromagnetic-induced-transparency (EIT)^17^,^18^. Besides requiring the capacity to store quantum information in QN nodes with long lifetime and high efficiency, routing the retrieved photon qubits from an atomic memory into one of many output channels is also needed. For example, in some quantum information protocols such as quantum repeater with multiplexed memories^19^,^20^ and scalable quantum computing with atomic ensembles^21^, the stored photons in atomic ensembles are required to be released into a desired quantum channel according to the outcome of a measurement^19^,^20^. By introducing optical switches into QNs, one can rout the retrieved photons into different spatially-separate photonic channels. However, the introduction of these optical switches will result in additional optical losses and also disturb the quantum states of the single photons. To avoid these shortcomings, one can integrate the function of optical switches into quantum memory. In the past decades, quantum node with the ability to route single photons into a desired channel have been theoretically proposed and experimentally demonstrated in various physical systems such as cavity-QED system^22^, circuit QED system^23^, opto-mechanical system^24^, waveguide-emitter system^25^,^26^,^27^,^28^, and pure linear optical system^29^ and so on^30^. For EIT-based light storage or slow-light systems, two- or three-channel optical routers of signal light fields have been realized by applying control light beams propagating along different directions^31^,^32^ or switching on reading light beams operating on different wavelengths^33^. Recently, in a gradient-echo memory system, spatially addressable readout and erasure of an image has been experimentally demonstrated^34^. However, since the input light signals in these experiments^30^,^31^,^32^,^33^,^34^ are intensive pulses, the quantum feature of these optical routers have not been characterized.

Here, we demonstrate an experiment in which the readouts of quantum memories for photonic polarization qubits (PPQs) can be routed into any one of multiple spatially-separate photonic channels. The PPQs are implemented with an arbitrarily-polarized coherent light (input signal) pulses at the single-photon level and co-propagate with a writing light beam through the cold atomic ensemble along x-axis. By means of EIT-based optical storage scheme^35^,^36^,^37^, i.e., turning off a writing beam, we store the input PPQs as spin waves (SWs) in the cold atoms. For obtaining long-lived memories for PPQs, we apply a bias magnetic field along x-axis to lift degenerate Zeeman sub-levels and then remove fast dephasing coming from magnetic-field-sensitive SWs out of the memories. Reading-beam light pulses propagating along a direction at a small angle respect to the quantum axis defined by the magnetic field are applied to release the stored PPQs into an output channel. By changing the read-beam propagating directions, we release the stored PPQ into 7 different output channels, respectively. At one of the output channels the measured maximum quantum-process fidelity for the retrieved single-photon polarization states is 94.2% for storage time of *δt* = 0.85 *ms*. At storage time of 6 ms, the quantum-process fidelity is ~80%, which is still beyond the threshold for the violation of the Bell inequality.

## Experimental setup

**Figure 1:**
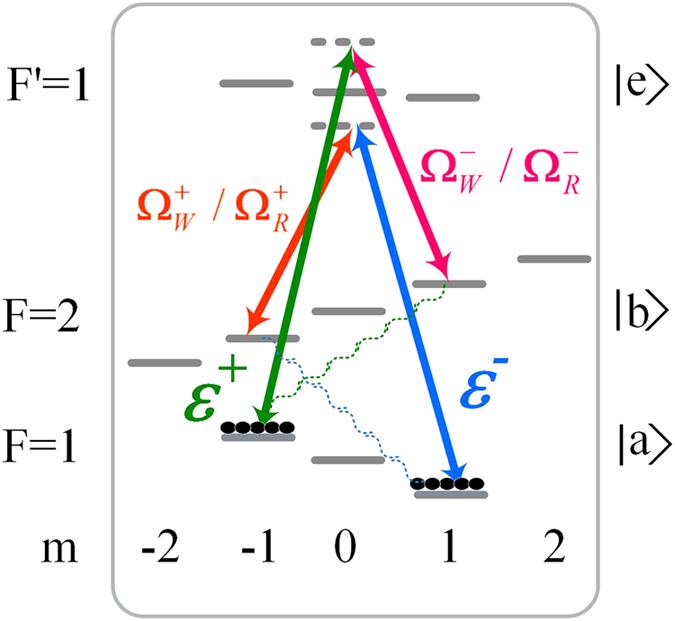
Three-level Λ-type EIT system for the storage of the signal fields in a moderate magnetic field (*B*_0_ = 12.5 *G*). *ε*^+^ and *ε*^−^ denote right-circularly and left-circularly polarized components of the signal light field, respectively. 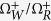 and 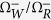 denote right-circularly and left-circularly components of the linearly polarized writing/reading light fields, respectively.

The involved levels of ^87^Rb atoms is shown in Fig. 1, where 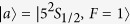, 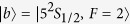 and 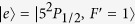. The frequencies of the input light signal and write/read coupling optical fields are tuned to the 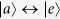 and 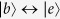 transitions, respectively, the frequency difference between them equals to Δ*ω* = *ω*_*be*_ − *ω*_*ae*_, which is consistent with the resonance of the two-photon 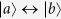 transition. PPQs are implemented with an arbitrarily-polarized coherent light (input signal) field 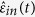 at single-photon level. The signal light field 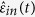 is expressed by:


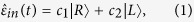


where 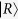 and 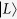 denote right-circularly (*σ*^+^) and left-circularly (*σ*^−^) polarized components, respectively, *c*_1_ and *c*_2_ are their amplitudes with |*c*_1_|^2^ + |*c*_2_|^2^ = 1. The write (read) optical field is vertical polarization and then can be viewed as the superposition of *σ*^+^- and *σ*^−^- polarized components 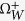 (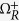) and 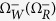. The quantum axis is determined by applying a magnetic field *B*_0_ along x-axis. The atoms are initially prepared into Zeeman sublevels 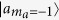 or 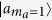 with equal population (*m* represents the magnetic quantum number) by optical pumping. By means of dynamic EIT process, i.e., turning off the write laser beam, we can store right-circular and left-circular polarization components of the PPQ as two distinct SWs. Under the condition of a weak bias magnetic field, both SWs include magnetic-field-sensitive and magnetic-field-insensitive SW components and thus storage lifetime is very short due to the fast decay of the magnetic-field-sensitive SWs. In order to eliminate the bad influences of the magnetic-field-sensitive SWs, we follow the storage scheme demonstrated in ref. 17, i.e., imposing a magnetic field of *B*_0_ = 12.5 *G* on a cold-atom ensemble to lift degenerate Zeeman sub-levels [as show in Fig. 1]. In this case, the magnetic-field-sensitive SW components are eliminated from EIT storage systems and the PPQ can be only mapped on two magnetic-field-insensitive SWs 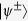, respectively. The magnetic-field-insensitive SWs 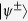 are associated with the coherences 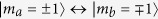, which can be expressed as:


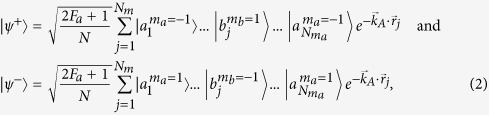


respectively, where 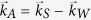 is wave-vector of the two SWs 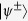, 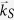 and 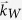 are the wave-vectors of the signal and write fields, respectively. To reduce the decoherence due to atomic random motion^13^, we align the input signal and write laser beams collinearly to propagate through the cold atom ensemble along x-axis, thus, 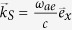, 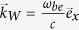, and the wavelength of SWs reach their maximal value 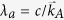, where 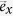 is the unit vector along x-axis.

**Figure 2:**
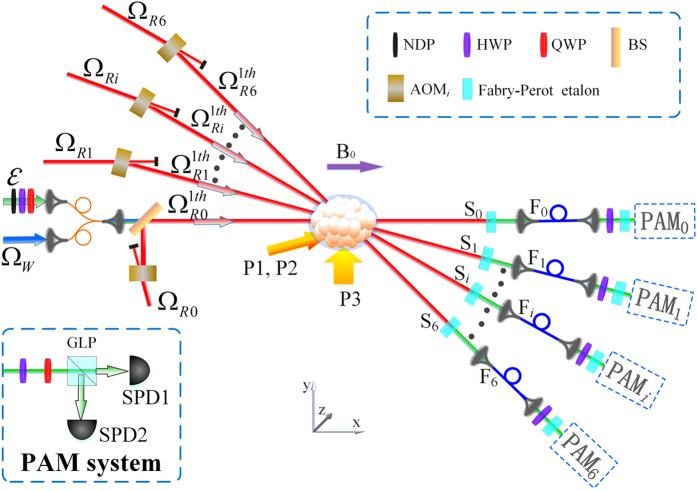
Experimental setup. P1 and P2: right-circularly and left-circularly polarized pump laser beam, respectively; P3: linearly polarized pump laser beam; *ε*: signal light beam; Ω_*W*_: writing light beam; 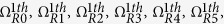 and 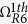: 1-th order diffractions of the reading beams Ω_*R*0_, Ω_*R*1_, Ω_*R*2_, Ω_*R*3_, Ω_*R*4_, Ω_*R*5_, which propagate along the directions at the angles of *θ* = 0°, *θ* = 0.4°, *θ* = 0.8°, *θ* = 2°, *θ* = 3°, *θ* = 4°, and *θ* = 5°, respectively; NDP: neutral density filters; FBS: fiber-beam-splitter; BS: polarization-insensitive beam splitter; AOM: acousto-optic modulator. *F*: single-mode optical fiber. GLP: the Glan-laser polarizer; PAM system: polarization analyzing and measuring (PAM) system, SPD: single-photon detector.

After a storage time *δt*, we apply a reading light pulse to convert the two stored SWs into flying PPQ. As shown in Fig. 2, a reading light field Ω_*Ri*_ propagates along the direction at a small angle of *θ* relative to *x*-axis, its wave-vector is written as: 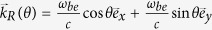, 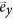 is the unit vector along *y*-axis. The wave-vector of the retrieved signal photons 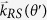 can be calculated according to the phase matching condition 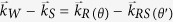
38, which is:


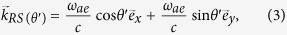


where the angle 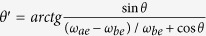. For ^87^Rb atomic system, we can calculate (*ω*_*ae*_ − *ω*_*be*_)/*ω*_*be*_ ≈ 10^−5^. So, for the case of *θ* << 5°, we have cos *θ* >> 10^−5^, thus *θ*′ ≈ *θ*, which means that the retrieved signal photons approximately propagate along the same direction as that of the reading beam and then we can effectively collect the retrieved signal photons along the reading-beam direction. Based on this fact, we can release the stored PPQ into a desired output port by selecting an appropriate reading-beam propagating direction around x-axis.

The experimental setup is shown in Fig. 2. The atomic ensemble is a cigar-shaped cloud of cold ^87^Rb atoms which is provided by a two-dimension (2D) magneto-optical trap (MOT). The size of the cold atom ensemble is about 4 mm × 4 mm × 7.5 mm. A 794.8-nm grating-feedback diode laser is used as sources of laser beams, which passes through two high-frequency AOMs. The two outputs from the AOMs obtain a frequency difference of Δ*ω* = *ω*_*be*_ − *ω*_*ae*_ = 6.834683 GHz and are used as the write/read coupling and input signal laser beams, respectively. We combine the input signal and write laser beams with a fiber beam splitter (FBS), which has a 95% (5%) transmission for the signal (writing) beam. Before arriving FBS, the signal light beam passes through neutral density filters, a λ/4 plate (QWP) and a λ/2 plate (HWP). The neutral density filters are used to reduce the intensity of the coherent signal light pulse. By adjusting QWP and HWP, we can obtain an on-demand polarization state of the signal field. After the FBS, the signal and write light beams collinearly propagate through the cold atoms along x-direction. The *σ*^−^- polarized pumping laser P1 and *σ*^+^- polarized pumping laser P2 collinearly propagate through the atoms with a deviation angle ~2° from x-direction, which drive the transitions 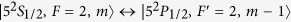 and 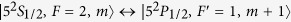, respectively. The π-polarization pumping laser P3 propagates through the atoms along y-direction, which drives the transition 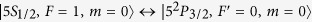. Due to optical pumping by the three pumping lasers, most of the atoms will be prepared into the Zeeman sublevels 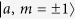 with an equal probability. The spot sizes (powers) of the P1, P2 and P3 laser beams in the center of cold atoms are ~7 mm (~10 mW), ~7 mm (~10 mW), 20 mm (~4 mW), respectively. We use several reading beams (Ω_*R*0_ … Ω_*Ri*_ … Ω_*R*6_) which respectively propagate along different directions to retrieve the stored PPQ. The switch-on of each reading beam Ω_*Ri*_ is controlled by an acousto-optic modulator (AOM_*i*_) and the first-order output 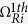 of the reading beam Ω_*Ri*_ from AOM_*i*_ is directed into the spatial mode (channel) *S*_*i*_ with the angle *θ*_*i*_ relative to the *x*-axis. The power of each reading beam is 15 mW, whose spot size is ~4 mm. When the reading beam light pulse 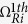 illuminates the atoms, the stored SWs are converted into the retrieved signal photons, which goes into the channel *S*_*i*_ and then is collected by a single-mode optical fiber *F*_*i*_. Before the optical fiber *F*_*i*_, a Fabry-Perot (FP) etalon is placed in the path to block the reading beam into the optical fiber *F*_*i*_. After the fiber *F*_*i*_, we utilize a HWP to compensate the phase difference between the retrieved right-circularly-polarized and left-circularly-polarized photons. Passing through the HWP, the retrieved photons passes through 4 FP etalons and then are sent to a polarization-measurement-and-analysis setup for observing polarization fidelity of the signal photon. The 5-FP-etalon transmission is 58% for the signal light and ~10^−13^ for the writing/reading light.

The experiments of storages and retrievals of the signal light field are carried out in a cyclic fashion with a repetition frequency of 20 Hz. In each cycle, the ^87^Rb atoms are trapped into the magneto-optical trap (MOT) for 42 ms. After which, we start to apply the bias magnetic field *B*_0_, whose value reaches 12.5 G during a time interval of 0.3 ms. Then pumping light beams P1, P2, P3 and the write light beam with a power of 2.5 mW are switched on. After a 5-μs optical pumping, half the atoms is prepared in the state 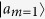 and half in the state 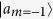. The measured optical depth of the 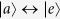 transition is ~10. At the initial time, i.e., *δt* = 0 *μs*, the signal light pulses (PPQs) with a 100-ns duration length are send out and transferred into the SWs in the cold-atom ensemble by turning off the write laser beam. After a delay time *t*, we transferred the stored SWs back into photon pulses by turning on the read beam 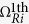 (*i* = 0,1... 6).

## Results

**Figure 3:**
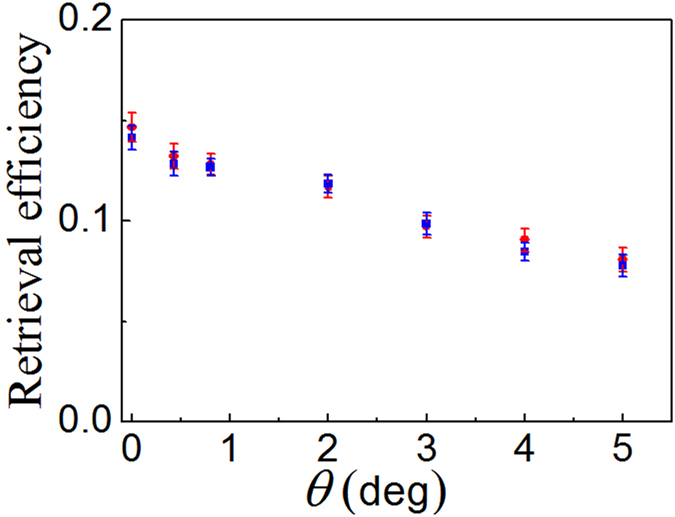
Measured retrieval efficiency of the signal light field versus the angle *θ* for a storage time of *δt* = 5 *μs*. The red-circle and blue-square dots are the experimental data of the *σ*^+^- and *σ*^−^- polarized input signal light, respectively.

First, we measure the retrieval efficiencies of the *σ*^±^- polarized components of the signal light field versus the angle *θ* for a storage time of *δt* = 5 *μs*. The measurements are carried out when the signal-beam input peak power is 25 μW and the retrieved signal pulses are detected by photodiode detectors. The red-circle and blue-square dots in Fig. 3 are the measured results of the *σ*^+^- polarized and *σ*^−^- polarized components of the signal light field, respectively. At *θ* = 0°, the retrieval efficiency reach to its maximum value *R*_*θ*=0°_(0) = 14%. With increasing in the angle *θ*, the retrieval efficiency *R*_*θ*_ decreases. For *θ* = 5°, the retrieval efficiency 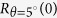 reduces to ~8%. We attribute such reduction to spatial-mode imperfect overlap (walk-off) between the stored SWs and the single-mode fiber *F*_*i*_, which increases as the angle *θ* increases.

**Figure 4:**
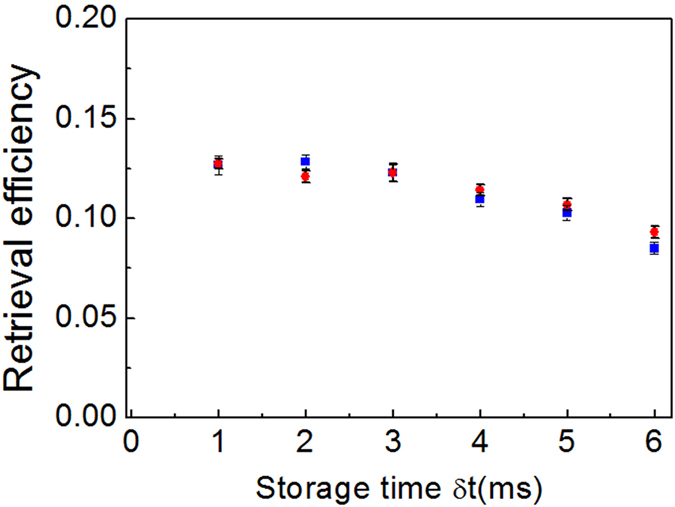
Measured dependence of retrieval efficiency on the storage time *δt* for *θ* = 0.8°. Red circular and blue square points are the measured results for the *σ*^+^- and *σ*^−^-polarized input signal light field, respectively. The black solid curve is the fit to the measured data based on the formula *R*_*θ*=0.8°_(*t*) = *R*_*θ*=0.8°_(0)exp(−*t*/*τ*) with *R*_*θ*=0.8°_(0) = 12.7%, yielding a memory lifetime of *τ* = 2.9 ms.

Next, we measure the time dependence of the retrieval efficiencies of the signal light fields at a fixed angle of *θ* = 0.8°. The red circle and blue square dots in Fig. 4 are the measured results of the right-circularly and left-circularly polarized signal light fields, respectively, and the black solid curve is the fit to the measured data based on the formula *R*_*θ*_(*t*) = *R*_*θ*=0.8°_(0)*e*^−*t*/*τ*^ with *R*_*θ*=0.8°_(0) = 12.7%, which yield a storage lifetime of ~2.9 ms. Such lifetime is longer than that (1.5 ms) in the previous work^17^. The main reason is that the magnetic-field gradient of ~35 mG in the previous work is reduced to 5 mG/cm in the presented work^17^.

Subsequently, we measure the polarization fidelities of the retrieved PPQ for several different angles *θ*. By using the neutral-density filters, we attenuated the signal light pulse into the single-photon level (i.e., the average photon number 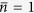)^17^. The total detection efficiency is *η*_*d*_ = 23%, which includes coupling efficiency of the single-mode fiber (80%), total transmission for the 5 Fabry-Perot etalons (58%), the efficiency of multi-mode fiber coupling to single-photon detectors SPD1 or SPD2 (97%) and the quantum efficiency of the single-photon detectors SPD1 and SPD2 (50%).

**Figure 5:**
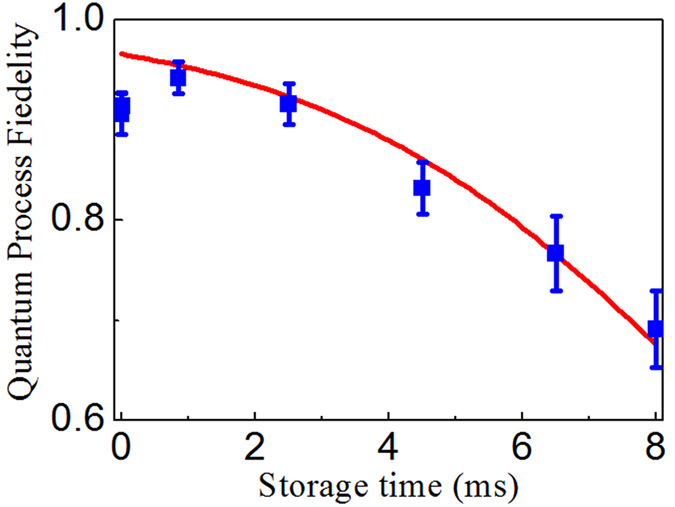
Measured time dependence of quantum process fidelity for *θ* = 0.8°. The red solid curve is the fitting to the data based on the Eq. [3] in ref. 17. The errors represent ±1 standard deviation, which are obtained from Poissonian detection statistic with Monte Carlo simulation.

The quality of storage and retrieval of PPQs may be judged by quantum process matrix *χ*, which can be obtained according to the relation^39^,^40^:


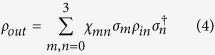


where *σ*_*i*_ are Pauli operators, *ρ*_*in*_ and *ρ*_*out*_ are the density matrixes of the input signal photons and retrieved signal photons, respectively. We follow the measurement method described in ref. 17 to obtain the density matrix *ρ*_*out*_. Based on the obtained density matrix *ρ*_*out*_, we reconstruct the matrix *χ* and then obtain quantum-process fidelity *F*_*Process*_ according to the definition 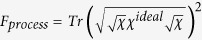 with 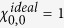
39. At a storage time of *δt* = 5*μs*, we measure the quantum-process fidelities *F*_*Process*_ for several different angles *θ*, the results are listed in Table 1, which show that they are not less than 89%. At *θ* = 0.8°, we measure the dependence of the quantum-process fidelity (QPF) *F*_*Process*_ on storage time *δt*, which is plotted in Fig. 5 (square dots). From the Fig. 5, one can see that the measured QPF decrease with storage time. At a storage time of *δt* = 6 *ms*, the measured value of QPF is still beyond 78%, which is consistent with that (4.5 ms) obtained in ref. 17, showing that the routing of the stored PPQs doesn’t shorten the coherent time for preserving PPQ.

## Discussion

In summary, we have demonstrated an experiment in which the long-lived quantum memories for photonic polarization qubits (PPQs) can be controllably routed into multiple spatially-separate photonic output channels, respectively. The measured quantum process fidelity of the retrieved PPQ for each of the channels is more than 89%. For a storage time of 6 ms, the measured process fidelity of the retrieved PPQs is still higher than the 78% bound to violate the Bell’s inequality^41^. The retrieval efficiency for a zero storage time is 14%, which can be further improved by either increasing the optical depth of the cold atoms^18^,^42^ or coupling the atoms into an optical cavity^16^,^43^. Base on the scheme that the stored PPQs can be controllably routed into different spatial channels without reducing their storage lifetime and retrieval efficiency in a small angle (*θ* ≤ 1°), one can build efficient quantum memory elements capable of routing retrieved photon qubits and then can find applications in quantum information processing based on quantum internet.

## Additional Information

**How to cite this article**: Chen, L. *et al*. Controllably releasing long-lived quantum memory for photonic polarization qubit into multiple spatially-separate photonic channels. *Sci. Rep.* **6**, 33959; doi: 10.1038/srep33959 (2016).

## Figures and Tables

**Table 1:** Quantum process fidelities *F*_*process*_ for different angles *θ* at a storage time of *δt* = 5 *μs*.

Angle	*θ* = 0°(*S*_0_ mode)	*θ* = 0.4°(*S*_1_ mode)	*θ* = 0.8°(*S*_2_ mode)	*θ* = 2°(*S*_3_ mode)	*θ* = 3°(*S*_4_ mode)	*θ* = 4°(*S*_5_ mode)	*θ* = 5°(*S*_6_ mode)
*F*_*process*_ (%)	90.2 ± 2.6	90.3 ± 1	91.4 ± 1.4	90.6 ± 2.3	91 ± 2	89.1 ± 1.8	89.5 ± 2.4

The error bars represent ±1 standard deviation, which are obtained from Poissonian detection statistic with Monte Carlo simulation.
